# Clinical and Electrocardiographic Predictors of Cardiac Resynchronization Therapy Response That Correlate with the 6 min Walking Test

**DOI:** 10.3390/jcm13206287

**Published:** 2024-10-21

**Authors:** Andrei Mihnea Rosu, Theodor Georgian Badea, Florentina Luminita Tomescu, Andreea Liana Rosu, Emanuel Stefan Radu, Oana Andreea Popa, Liliana Catalina Andrei, Crina Julieta Sinescu

**Affiliations:** 1 Department of Cardiology, Prof. Dr. Agripa Ionescu Emergency Hospital, 077015 Bucharest, Romania; andrei-mihnea.rosu@drd.umfcd.ro (A.M.R.); radu.emanuel@dcti.ro (E.S.R.); popa.oana@dcti.ro (O.A.P.); 2 Department of Anatomy, Doctoral School of Carol Davila University of Medicine and Pharmacy, 022328 Bucharest, Romania; 3 Department of Radiology, Prof. Dr. Agripa Ionescu Emergency Hospital, 077015 Bucharest, Romania; 4 Department of Radiology, Carol Davila University of Medicine and Pharmacy, 022328 Bucharest, Romania; 5 Department of Clinical Pharmacology, BBraun, 013714 Bucharest, Romania; andreea.rosu@bbraun.com; 6 Department of Cardiology, Bagdasar-Arseni Emergency Hospital, Carol Davila University of Medicine and Pharmacy, 022328 Bucharest, Romania; catalina.andrei@umfcd.ro (L.C.A.); crina.sinescu@umfcd.ro (C.J.S.)

**Keywords:** cardiac resynchronization therapy, 6MWT, six-minute walk test, heart failure, QRS area, QRS duration, intraprocedural blood pressure, exercise capacity, electrocardiography

## Abstract

**Background:** Cardiac resynchronization therapy is an essential treatment for heart failure patients. Candidates typically have cardiomyopathy accompanied by delayed electrical activation in the left ventricular lateral wall, causing uncoordinated contractions and worsening heart failure. Heart failure severity can be assessed with functional tests: the cardiopulmonary test, which is a maximal exercise test, remains the gold standard, but the 6 min walk test has emerged as an easier, faster, and more comfortable alternative to be used by clinicians to adjust treatment protocols for cardiovascular and pulmonary conditions. **Methods:** This is a prospective observational study that included 69 patients from a single healthcare facility, and the purpose was to determine if the 6 min walk test results could be associated with changes in various electrocardiographic, clinical, functional, and demographic parameters. All the parameters and the 6 min walk distance were recorded at four key time moments: before the procedure and after 6, 9, and 12 months. The electrocardiographic parameters were obtained from the patients’ electrocardiograms recorded in the four key moments and included variables such as QRS area, duration, percentage of biventricular pacing, and many others, while the functional variables included the monitored intraprocedural systolic blood pressure and the end-systolic left ventricular volume. We also aimed to check if clinical conditions such as diabetes and chronic kidney disease and demographic variables such as age or sex have any impact. **Results and Conclusions:** All this research was performed in order to identify which parameters hold a predictive value and can serve as future criteria for better patient selection and for defining a proper resynchronization outcome. The study shows that parameters such as diabetes and QRS duration have an impact over the 6 min walk distance. Also, newer variables such as the QRS area and the R/S ratio may represent a direction worth studying in order to predict the outcomes of cardiac resynchronization therapy.

## 1. Introduction

The six-minute walk test represents a functional testing modality designed to measure the exercise capacity of patients with cardiopulmonary diseases [1]. It provides a comprehensive evaluation of the various systems engaged during physical activity and has the advantage of being a simple, inexpensive, and straightforward test that helps determine the extent of functional impairment [2]. The test is performed by having the subjects walk on a flat, hard surface (a corridor of 40 m) for six minutes (hence the alternate name of 6 min walk distance; 6MWD). The patient is advised to remain calm, take their medications, and wear comfortable clothing and shoes. The supervisor records baseline oxygen saturation measurements, heart rate, brachial arterial blood pressure, and the Borg scale rating for dyspnea and fatigue. Once the patient understands the instructions, they can begin the test. The walking course should be marked every 3 m, with cones placed at the turnaround points. During the test, participants should walk at a pace appropriate for their condition, and they are allowed to stop or slow down if necessary, resuming walking as soon as possible. The supervisor remains present throughout the test, encouraging the patient [3]. At the end of the test, the supervisor once more records the Borg scale ratings for dyspnea and fatigue. Optionally, arterial blood pressure, heart rate, and oxygen saturation can also be measured again. The number of laps completed and any additional distance covered are recorded, and the total 6 min walk distance (6MWD) is calculated.

It is well known that cardiac resynchronization currently represents one of the best treatment options for patients with heart failure caused by an electric substrate, but its implementation is riskier than other implantable electronic devices, and approximately 30–50% of patients receiving CRT do not benefit from the treatment and are classified as non-responders [4,5,6]. Until now, many studies aimed to identify clinical and echocardiographic parameters that predict responsiveness to CRT and asses functional improvement and echocardiographic remodeling, but the exact determinants of mortality are still poorly understood [7]. The 6-minute walk test (6MWT) is an accessible and well-tolerated method for assessing the functional capacity of patients with heart failure (HF). Although the cardiopulmonary exercise test is the gold standard for evaluating exercise capacity in HF patients, the 6MWT provides valuable insight into a patient’s daily activity levels. In our paper, we aim to highlight the significance of the 6MWT in HF patients, exploring its usefulness, limitations, and association with various parameters during cardiac resynchronization therapy. Additionally, we aim to examine the test’s prognostic role and how it reflects changes following CRT in HF patients [8,9].

## 2. Materials and Methods

### 2.1. Ethical Approval

The study was conducted as a unicenter observational prospective research project in the Department of Cardiology at the Prof. Dr. Agrippa Ionescu Emergency Clinical Hospital in Balotesti, Ilfov, Romania, spanning from 2017 to 2022. The Ethics Committee of the Carol Davila University of Medicine and Pharmacy in Bucharest, Romania (15037/5 June 2017), approved the study protocol. The planning, data collection, and reporting involving human subjects were in accordance with the principles of the Declaration of Helsinki. Written informed consent was obtained from all participants following General Data Protection Regulation (GDPR) requirements.

### 2.2. Study Design

The study is a prospective observational investigation involving 69 individuals diagnosed with cardiac failure and dilated cardiomyopathy presenting with a low ejection fraction (FE) and significant left bundle branch block (LBBB). Data for this analysis were extracted from patient records at Agrippa Ionescu Hospital in Balotesti, Ilfov, Romania. The study included patients who underwent implantation of a cardiac resynchronization therapy (CRT) device between 2017 and 2022. Baseline information was retrieved from the hospital’s patient information system, encompassing details on heart failure etiology, comorbidities, and medication histories obtained from patient records and pertinent medical documentation. Regarding the sample size for this paper, it is important to keep in mind that this study was performed in a single healthcare facility, and the cardiac resynchronization procedures were performed by a single interventional cardiologist. Also, one should keep in mind that the country where the study was performed is a relatively small country, and the healthcare system is designed to provide healthcare to all the citizens. By doing so, there is a need to provide funds for all the specialties in an equal manner (and in the last years, the funding has faced some challenges due to some well-known economic and geo-political conditions in the eastern part of Europe). Finding patients with cardiac failure suitable for resynchronization therapy is particularly challenging in a country with a relatively small population, where the prevalence of eligible cases is naturally lower. Moreover, as minimally invasive procedures are still emerging in this setting, the pool of candidates is further limited. Despite these challenges, identifying and treating even a small sample of patients is highly relevant. Each successful case contributes valuable data and clinical experience, helping to refine treatment protocols and expand access to this life-saving therapy in the future. Such facts do not constitute an advantage, and the present paper also acts as a reminder for other professionals willing to perform research in underfunded healthcare systems that it will be a difficult task, but not getting discouraged and overcoming drawbacks is the key to improvement.

#### 2.2.1. Inclusion Criteria

For this current analysis, patients were deemed eligible for CRT device implantation based on predefined inclusion criteria, including symptomatic heart failure with left bundle branch block (LBBB) QRS morphology, a baseline QRS duration exceeding 130 milliseconds, and an ejection fraction (EF) less than 35% despite receiving optimal medical therapy. The inclusion criteria and the patient treatment at the beginning of the study were in accordance with the 2016 ESC guidelines for the diagnosis and treatment of acute and chronic heart failure and were updated to the newer 2021 version [10,11].

#### 2.2.2. Exclusion Criteria

Patients with acute myocardial infarction or unstable angina (during the acute phase), uncontrolled arrhythmias causing symptoms or hemodynamic instability, acute myocarditis or pericarditis, uncontrolled acutely decompensated heart failure (such as acute pulmonary edema), acute pulmonary embolism, suspected dissecting aortic aneurysm, severe hypoxemia at rest, uncontrolled chronic obstructive pulmonary disease, or acute respiratory failure were excluded due to these being absolute contraindications for the 6MWT [9]. Additionally, patients with acute noncardiopulmonary disorders that might affect exercise performance or be exacerbated by exercise (such as infection, renal failure, or thyrotoxicosis) or those with mental impairment were also excluded.

### 2.3. 6MWT—The Outcome Variable

The 6MWT was performed in the hallway of the cardiology ward, which is about 60 m long. We established a 40 m distance for a lap and marked every 2 m. We made sure the subjects understood how to perform the test, and baseline oxygen saturation, heart rate, brachial arterial blood pressure, and the Borg scale rating for dyspnea and fatigue were noted for each patient. Three supervisors were present for the test, two at each end and one at the middle of the pathway. The supervisors ensured everybody had comfortable hospital clothes and shoes, guided the patients, and encouraged them during the test. When the 6 min timer ran out, the supervisors would note the total walked distance and perform another measurement for heart rate, oxygen saturation, blood pressure, and the Borg scale for fatigue and dyspnea.

The study also included baseline 12-lead ECGs and post-implantation paced 12-lead ECGs at 6, 9, and 12 months after implantation. Patient selection, device implantation, and follow-up procedures adhered to local protocols during enrollment. Patients received a Doppler ultrasound as screening for peripheral artery disease, but no significant stenoses were identified. Also, the diabetic patients included in our study did not complain of lower limb intermittent claudication during the 6 min walk tests. The outcome analyzed was the evolution of the 6 min walk test, assessed before the procedure and at three post-procedure intervals (6, 9, and 12 months).

### 2.4. Electrocardiographic Data and Intraprocedural Systolic Blood Pressure

The study included an ECG analysis before the procedure and at the 6-month, 9-month, and 12-month follow-up visits. Electrocardiographic data were obtained from paper electrocardiograms, which were scanned using a multifunctional scanner. Parameters such as different durations and amplitudes were measured on the electrocardiograms, and for the QRS area, we calculated the median value of the areas under the QRS complex graph. During each cardiac resynchronization procedure, arterial blood was continuously monitored using a 4 or 5Fr radial or femoral catheter to assess immediate changes after starting biventricular pacing. This aimed to validate improvements in cardiac output from correcting myocardial dyssynchrony.

### 2.5. Statistical Analysis

The measurements were recorded in our database and subjected to rigorous statistical analysis using R software 4.3.3 [12].

We employed a repeated measures ANOVA to determine the statistical significance of the observed differences. The ANOVA test is essential when evaluating the effects of different treatments or interventions, as it provides a clear and efficient way to analyze complex data sets, and it is particularly useful because it allows for the comparison of multiple groups simultaneously, determining whether there are statistically significant differences between their means. ANOVA works well with small samples because it effectively controls for type I errors, which can occur when making multiple comparisons, and ensures that any observed differences are not due to random chance, and this is why we considered it the right tool for deriving meaningful conclusions even for a limited sample size.

Subsequently, a post hoc analysis was conducted using paired Bonferroni *t*-tests to explore detailed specific comparisons. The Bonferroni was an excellent choice for post hoc analysis because it provided control for the error rate when making multiple comparisons. By adjusting the significance threshold, it reduced the likelihood of false positives, ensuring that any statistically significant differences identified were meaningful. It was particularly valuable in our study where the risk of drawing incorrect conclusions from small sample sizes has to be taken into account. For our study, the Bonferroni test confirmed that the observed differences in the 6 min walk test values across all follow-up time points were not only statistically significant but also reliable.

A linear mixed-effects model was used to identify factors that are associated with the evolution of the patients’ 6 min walk test value, with the dependent variable being the ejection fraction value tracked at 4 points in time; the independent variables (fixed effects) were the demographic, clinical, and electrocardiographic parameters tracked in the study; and the random effects (varying effects) were the unique intercepts for each patient in the study. In this study, the significance level (α) was set at 0.05, where *p*-values less than 0.05 were considered statistically significant.

**Figure 1:**
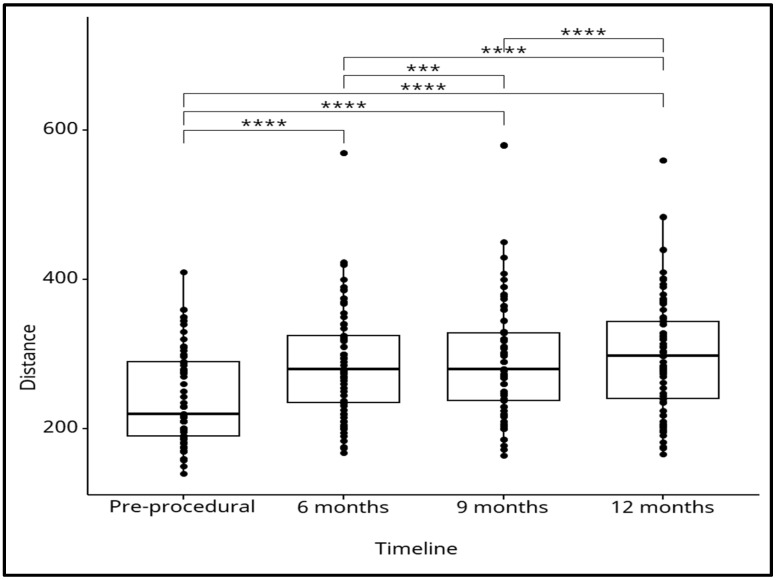
This figure shows the results of an ANOVA (Analysis of Variance) test comparing walking distances measured at different time points: pre-procedural, 6 months, 9 months, and 12 months post-procedural. Each boxplot represents the distribution of walking distances (likely from a six-minute walk test) at these intervals, with the median represented by the horizontal line inside the box, and the range of distances reflected by the whiskers and individual data points. The asterisks above the boxplots represent the significance levels of the differences between the groups being compared in the ANOVA post-hoc analysis. Each number of asterisks corresponds to a specific *p*-value range, indicating how statistically significant the difference is between the means of the groups: ***: *p* < 0.001 (very highly significant); ****: *p* < 0.0001 (extremely significant).

The 6 min walking distance (6MWD) was measured before the procedure and at each follow-up visit during a 12-month period for every patient that had undergone cardiac resynchronization therapy, and comparisons between different time points were conducted. By doing so, the 6 min walking distance (6MWD) was measured at four intervals: pre-procedural and at 6, 9, and 12 months post-procedural. Initially, the mean distance was 240.6 m, with a standard deviation of 63.5 m. By 6 months post-procedure, the mean distance increased to 286.7 m (SD: 72.5 m). Continued improvement was observed at the 9-month mark, with a mean distance of 291 m (SD: 75.2 m), ultimately reaching a peak of 297.2 m (SD: 77.2 m) at the 12-month follow-up. The ANOVA test was conducted to assess the statistical significance of the differences in the 6 min walking distances across the follow-up period. The results of the test, F(1.1, 74.36) = 82.10, *p* < 0.0001, indicate that the differences in mean distances covered at the various time points were statistically significant, confirming that the observed improvements over time were unlikely to be due to chance. Further, the post hoc analysis using paired Bonferroni *t*-tests confirmed statistically significant differences in the 6 min walking distance values across all follow-up time points. However, we observed that the most clinically significant differences were between the initial (pre-procedural) value and the values at each subsequent follow-up interval, and that suggested to us that while all measurements showed statistical significance, the most meaningful clinical improvements occurred in comparison to the baseline measurement (Figure 1).

Key observations:

- Pre-procedural vs. post-procedural: There is a significant improvement in walking distance at 6, 9, and 12 months compared to the pre-procedural measurements. The asterisks (****) denote statistically significant differences, indicating that the improvement in walking distance at each post-procedural time point is highly significant (*p* < 0.0001).
- Post-procedural time points: There are also significant differences between 6, 9, and 12 months, suggesting that the walking distance continues to improve or stabilize over time following the procedure. The increasing number of asterisks indicates highly significant improvements across these time points.
- Spread of data: Each boxplot shows some variation in walking distances, with outliers present at each time point (individual dots above the whiskers). This suggests that, while the majority of patients experienced improvement, there is some variability in individual responses.

To summarize, this figure demonstrates that the procedure resulted in statistically significant improvements in walking distance over time, with continued progress up to 12 months. The ANOVA test confirms that these changes are not due to random variation, but are meaningful improvements in patient outcomes.

### 2.6. Correlation Between Various Parameters and the 6 min Walk Test at 12 Months

In this research, we investigated several factors that could influence the 6 min walk test (6MWT) distance over a 12-month period, focusing on correlations between these parameters and walking performance. These parameters are included in Table 1, and each determined *p*-value can be seen in the last column. Each parameter was tested for the null hypothesis to determine whether or not it had any effect on the 6 min walking distance. As stated in the Methods section, the threshold for the *p*-value was set to 0.05, and a lower *p*-value (less than 0.05) suggested that the observed results were unlikely to have occurred by chance, leading us to reject the null hypothesis and consider the respective parameter significant for the evolution of the 6 min walking distance. Further on, the parameters that were considered significant were analyzed in concordance with the evolution of the 6 min test walking distance.

Among the primary parameters examined was age, based on 276 measurements. The results showed a slight negative correlation with 6MWT distance, but this relationship was not statistically significant (*p* = 0.267), suggesting that age alone may not be a strong predictor of walking performance in this cohort. Gender differences were also explored, with data from 11 females and 57 males. Males tended to walk further than females, though the correlation was not significant (*p* = 0.391), indicating that while gender-related differences in physical performance exist, other factors likely play a more substantial role.

Demographic factors, including rural versus urban residency, were also analyzed. Urban residents exhibited a non-significant trend toward shorter walking distances compared to their rural counterparts (Beta = −23, *p* = 0.174), suggesting potential environmental or lifestyle influences on exercise capacity, though further research is needed to draw firm conclusions.

Hemoglobin levels were measured at each visit, revealing a borderline positive correlation with walking distance (Beta = 10, *p* = 0.091). This suggests that higher hemoglobin levels may enhance oxygen transport and endurance, although the results were not statistically conclusive.

**Figure 2:**
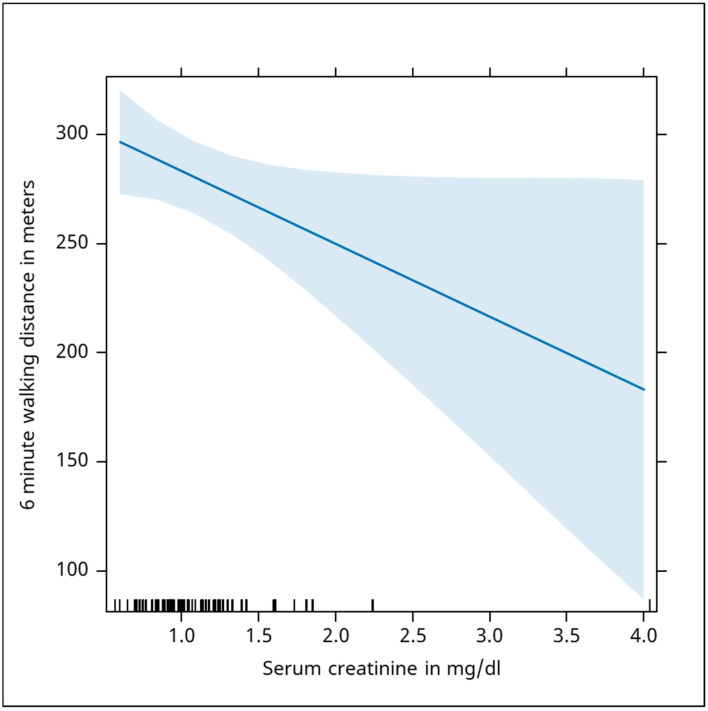
The chart illustrates a negative association between serum creatinine levels and the 6 min walking distance, indicating that higher creatinine levels are linked to reduced physical performance. The shaded area represents the confidence interval, showing variability in the data. The blue shaded area in the chart represents the confidence interval for the regression line, which shows the relationship between serum creatinine levels (in mg/dL) and the 6-minute walking distance (in meters). The confidence interval gives an estimate of the uncertainty or variability around the regression line. It shows the range within which we expect the true regression line to lie with a certain degree of confidence (in this case 95%). A narrower blue shaded area indicates less variability and more precision in predicting the relationship between serum creatinine levels and walking distance. A wider blue shaded area suggests greater variability, meaning the predictions become less precise as the serum creatinine levels increase.

Conversely, creatinine levels, measured 276 times, demonstrated a significant negative correlation with 6MWT distance (Beta = −33, *p* = 0.05), indicating that impaired renal function negatively impacted physical performance. The analysis has shown us that serum creatinine was negatively associated with the evolution of the 6 min walking distance, where an increase of 1 mg/dl was associated with an average distance decrease of 33 m (Figure 2).

**Figure 3:**
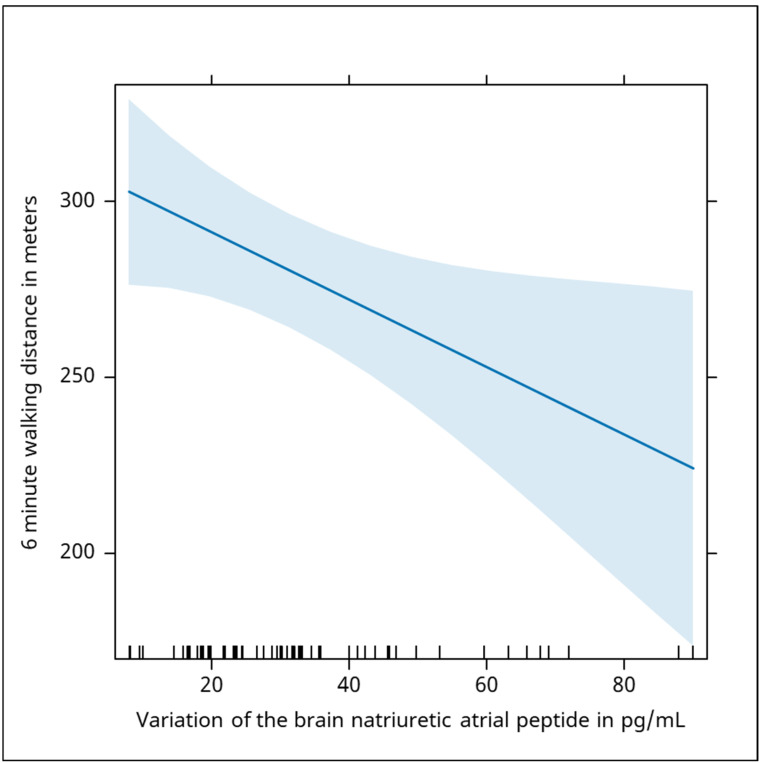
The chart shows a negative association between brain natriuretic atrial peptide (BNP) levels and the 6 min walking distance. As BNP levels increase, the average walking distance decreases, suggesting that higher BNP levels are linked to a reduced exercise capacity. Specifically, an increase of 100 pg/mL in BNP corresponds to an approximate decrease of 0.96 m in walking distance. The blue shaded area shows how the variability in the data affects the prediction of the 6-min walking distance at different BNP levels. The narrower portion of the blue area indicates more confidence in the predicted values at lower BNP levels. The wider portion suggests more variability or less precision in the predictions as BNP levels increase.

Additionally, NT-proBNP (brain natriuretic atrial peptide) levels showed a significant negative correlation with walking distance (Beta = −0.96, *p* = 0.028), highlighting the association between elevated cardiac biomarkers and reduced exercise capacity, emphasizing the role of cardiac function in physical performance. The value of the brain natriuretic atrial peptide was negatively associated with the evolution of the 6 min walking distance, with an increase of 100 in the serum value of the peptide being associated with an average decrease in the walking distance by 0.96 m (Figure 3).

**Figure 4:**
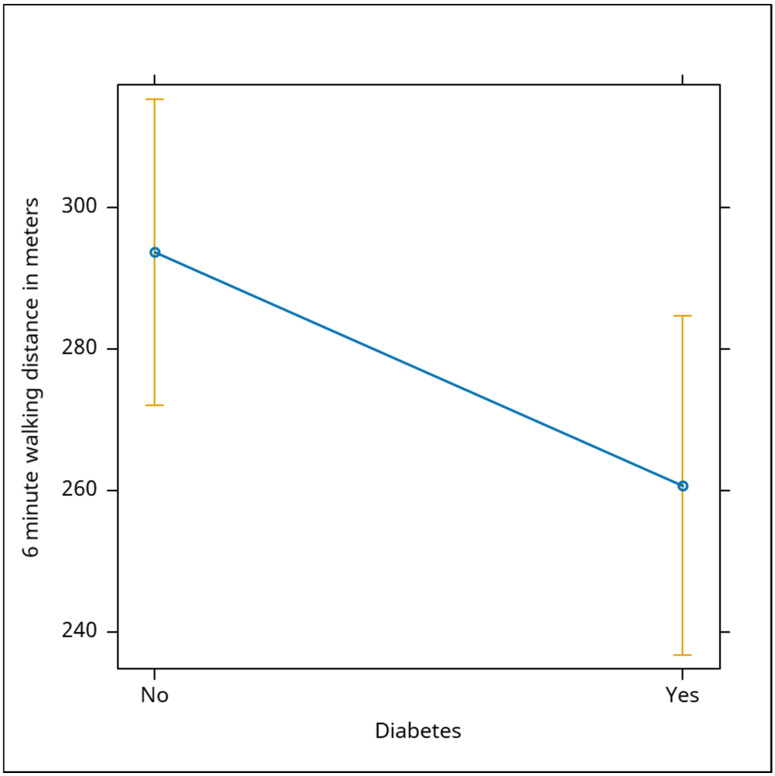
The chart highlights the impact of diabetes on the 6 min walking distance. There is a significant negative correlation between diabetes and walking distance, with diabetic patients covering, on average, 33 m less than non-diabetic individuals. This suggests that diabetes is associated with a reduction in exercise capacity. The yellow bars indicate variability within each group.

In the study we also examined the impact of ischemia and atrial fibrillation on the 6 min walk test (6MWT) distance, and found non-significant negative correlations with walking performance for both conditions (Beta: −25, *p* = 0.141 for ischemia; Beta: −24, *p* = 0.156 for atrial fibrillation). Although these heart conditions might affect exercise capacity, their influence appears complex and multifactorial. Diabetes, however, showed a significant negative correlation with walking distance (Beta: −33, *p* = 0.049), indicating to us that diabetic patients tended to have a reduced exercise capacity. The distance traveled during the 6 min walk test by patients with diabetes was shorter by 33 m compared to subjects without diabetes (Figure 4).

**Figure 5:**
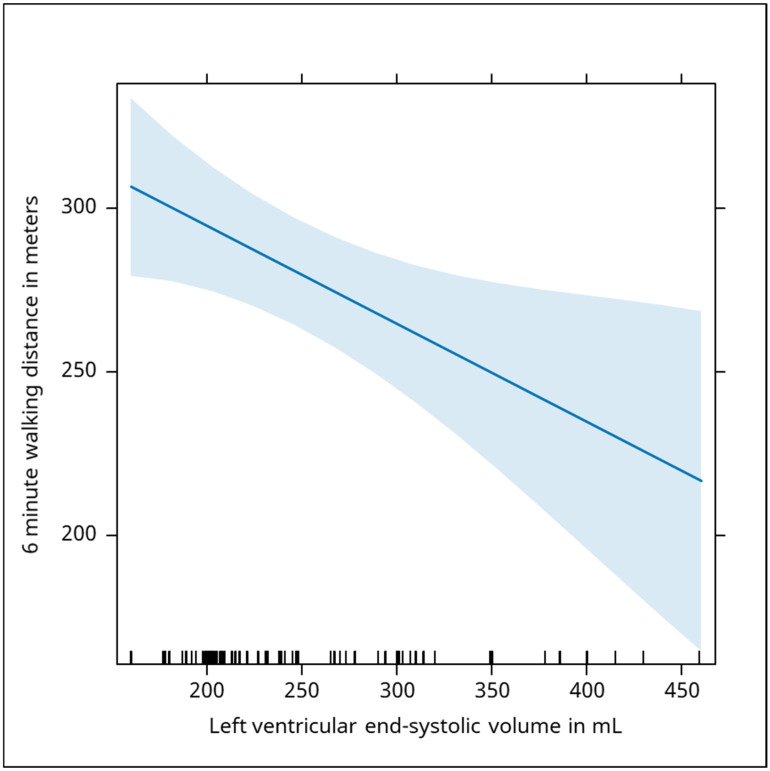
The graph shows a negative correlation between left ventricular end-systolic volume (LVESV) in milliliters and the distance covered in the six-minute walking test, measured in meters. The trend line indicates that as the LVESV increases, the walking distance decreases, emphasizing that worsening heart function (an increased LVESV) is associated with lower physical performance in the walking test. The blue shaded area represents the confidence interval around the regression line that illustrates the relationship between left ventricular end-systolic volume (LVESV) and the 6-min walking distance. A narrower interval (closer to 200–300 mL LVESV) indicates more confidence in the predicted walking distance in this range. A wider interval at higher LVESV values (350–450 mL) suggests more variability and less certainty in the predictions of walking distance as LVESV increases.

Additionally, left ventricular end-systolic volume (LVESV) was analyzed 276 times, and revealed a significant negative correlation with walking distance (Beta: −0.30, *p* = 0.016). LVESV was negatively associated with the distance traveled by the subjects. An increase of 1 mL in the LVESV was associated with a median drop of 0.30 m in the distance walked (Figure 5).

**Figure 6:**
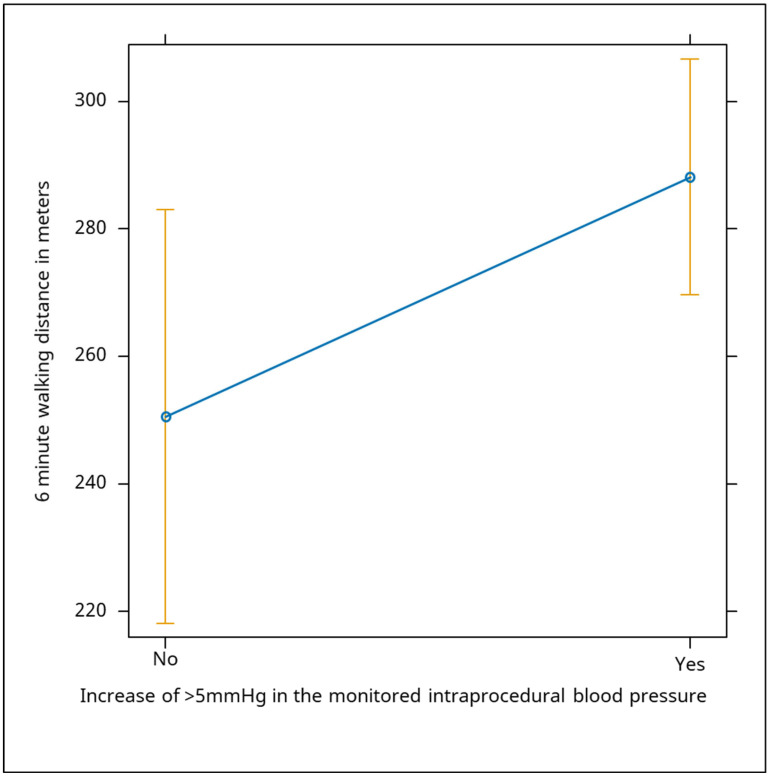
The graph shows the relationship between an increase of more than 5 mmHg in monitored intraprocedural systolic blood pressure and the distance covered in a six-minute walking test. The data demonstrate that patients who experienced an increase of more than 5 mmHg in systolic blood pressure during the procedure had a significant improvement in walking distance. The plot indicates a positive correlation (Beta: 38, *p* = 0.05), with a median increase of 38 m in walking distance for those with elevated intraprocedural blood pressure, suggesting that an increase in systolic blood pressure may be associated with enhanced exercise capacity, possibly due to improved systolic heart function.

Intraprocedural systolic blood pressure monitoring showed that patients with an increase of more than 5 mmHg had a significant positive correlation with walking distance (Beta: 38, *p* = 0.05), suggesting that improved systolic function may enhance exercise capacity. For the intraprocedural monitored systolic blood pressure, an increase in the monitored blood pressure over 5 mmHg was positively correlated with distance, the median increase being 38 m (Figure 6).

**Figure 7:**
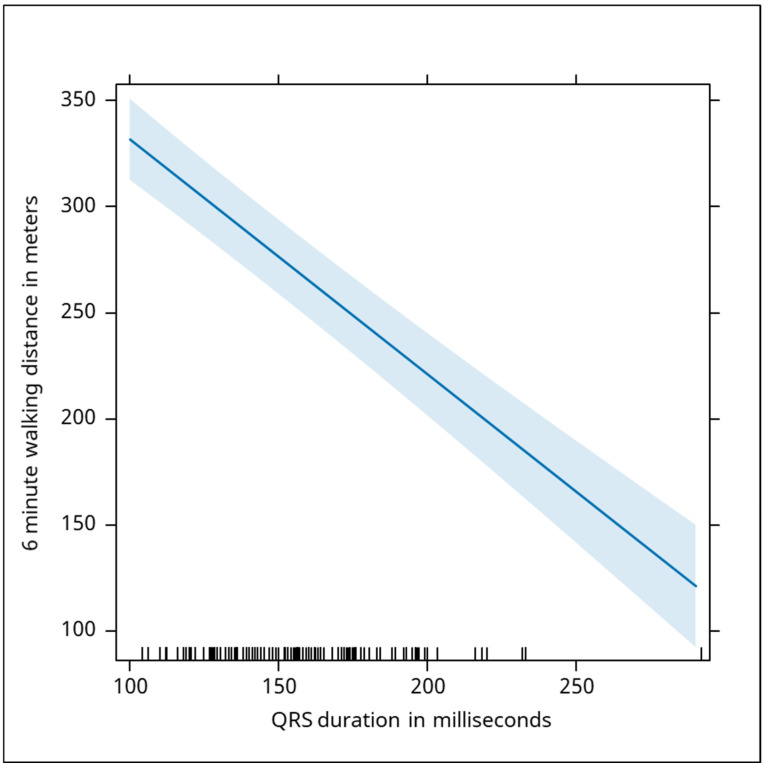
The graph illustrates a negative correlation between QRS duration in milliseconds and the six-minute walking distance in meters. As the QRS duration increases, the walking distance decreases. Specifically, a reduction of 1 millisecond in QRS duration is associated with an increase of 1.1 m in walking distance. This suggests that longer QRS durations, which may indicate delayed or abnormal electrical conduction in the heart, are associated with poorer physical performance as measured by walking distance. The shaded area around the trend line represents the confidence interval, and the tick marks along the bottom axis indicate individual data points of QRS duration. The blue shaded area represents the confidence interval around the regression line, indicating the uncertainty in predicting walking distance based on QRS duration. A narrower interval reflects more precise estimates, while a wider interval indicates greater variability in the predictions. The confidence interval in this graph is relatively narrow. This indicates that the relationship between QRS duration and the 6-min walking distance is precise and reliable across the range of values.

**Figure 8:**
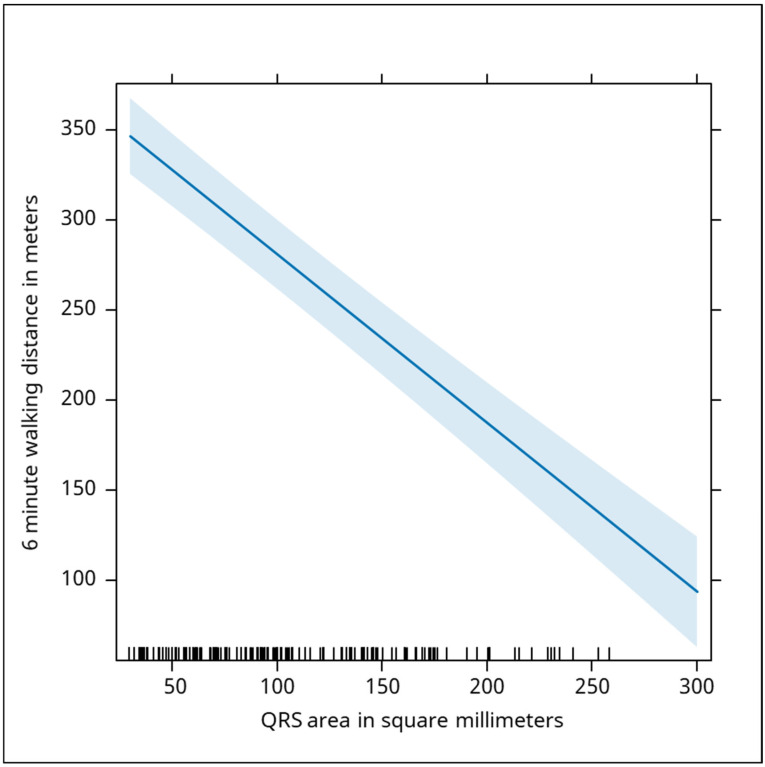
The graph demonstrates a negative correlation between the QRS area (measured in square millimeters) and the distance covered during the six-minute walking test (measured in meters). As the QRS area increases, the walking distance decreases. Specifically, each increase of 1 square millimeter in the QRS area is associated with a decrease of 0.94 m in walking distance. This suggests that a larger QRS area, which also indicates electrical or structural abnormalities in the heart, correlates with diminished physical performance. The blue shaded area in this graph represents the confidence interval around the regression line, showing the precision of the relationship between QRS area and 6-min walking distance. The interval is relatively narrow, indicating a consistent and reliable negative correlation between the two variables. As the QRS area increases, the walking distance predictably decreases with minimal variability in the data.

The electrical activity of the heart was assessed by QRS duration and QRS area, and the statistical tests demonstrated highly significant negative correlations with walking distance (QRS duration Beta: −1.1, *p* < 0.001; QRS area Beta: −0.94, *p* < 0.001). The duration of the QRS complex was negatively associated with the distance recorded. A drop of 1 millisecond in the duration was associated with an increase in the distance by 1.1 m (Figure 7). The QRS area was negatively correlated with the distance traveled by the subjects who performed the 6 min walk test. Every increase by 1 square mm was associated with a drop of the distance by 0.94 m (Figure 8).

**Figure 9:**
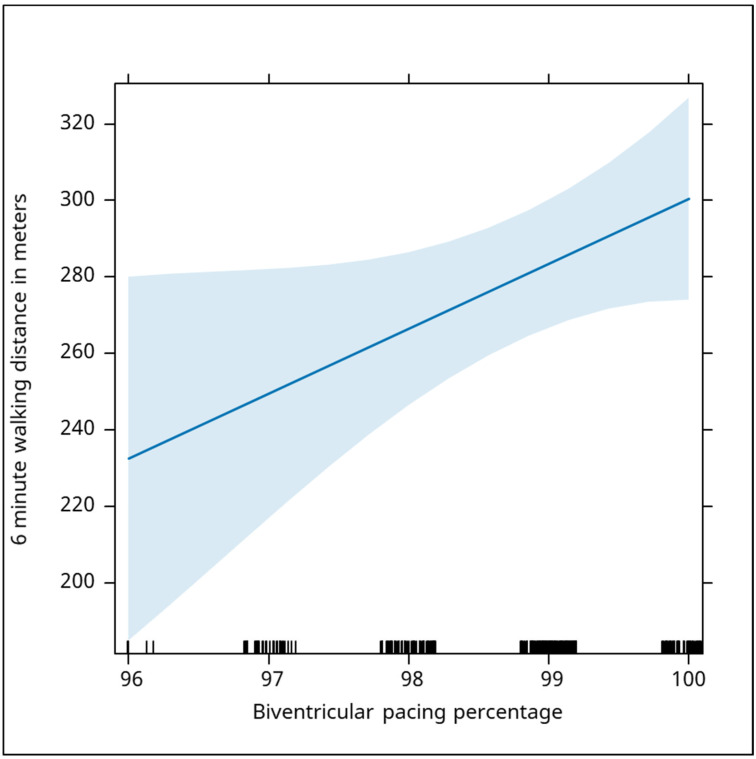
The graph demonstrates a positive correlation between the percentage of biventricular (BIV) pacing and the distance covered in the six-minute walking test. As the percentage of BIV pacing increases, the walking distance also increases. Specifically, each 1% increase in BIV pacing is associated with a median increase of 17 m in walking distance (Beta: 17, *p* = 0.046). This indicates that effective biventricular pacing has a significant positive effect on walking performance, with higher pacing percentages correlating with greater physical endurance as measured by walking distance. In this graph, the blue shaded area represents the confidence interval around the regression line, illustrating the relationship between biventricular pacing percentage and 6-min walking distance. The confidence interval is wider compared to previous graphs, indicating more variability in the data. While there is a positive correlation between higher pacing percentages and increased walking distance, the broader interval suggests less precision in the prediction, especially at higher pacing percentages.

Other electrical markers, such as R amplitude in the aVR and right precordial leads, did not show significant correlations (Beta: 3.3, *p* = 0.405; Beta: 7.3, *p* = 0.294, respectively). However, the percentage of biventricular (BIV) pacing exhibited a significant positive correlation with walking distance (Beta: 17, *p* = 0.046), indicating that effective pacing may play an important role in enhancing exercise capacity. From the analysis, an increase of 1 percent in the biventricular pacing percentage was correlated with a median increase in the distance of 17 m (Figure 9).

**Figure 10:**
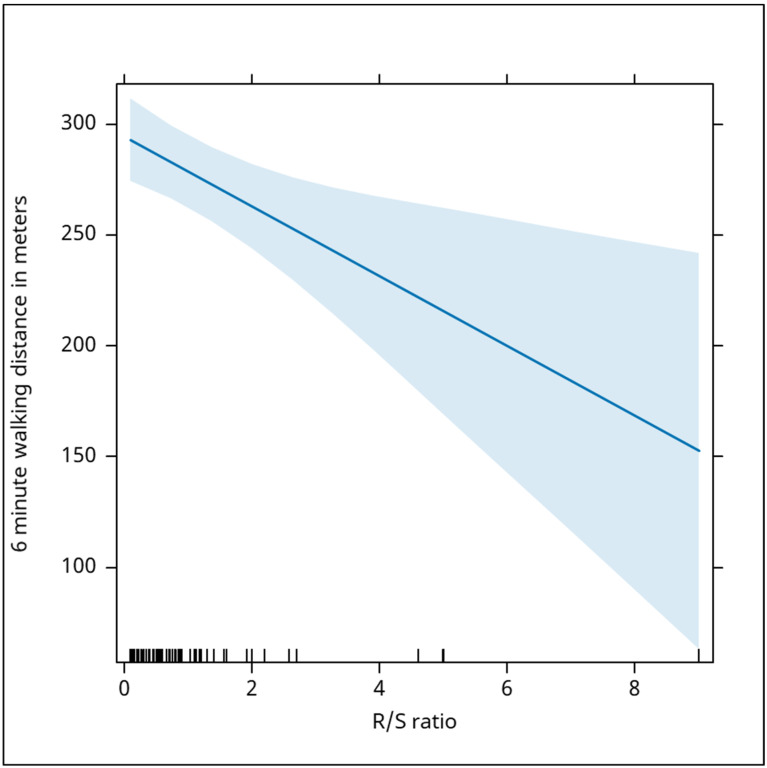
The graph illustrates a significant negative relationship between the R/S ratio and the distance covered in the six-minute walking test. As the R/S ratio increases, the walking distance decreases. Specifically, an increase of 1 in the R/S ratio is associated with a decrease of 16 m in walking distance (Beta: −16, *p* = 0.006), indicating that a higher R/S ratio, which could reflect certain cardiac electrical or functional abnormalities, is correlated with poorer physical performance, as evidenced by a shorter distance walked. The blue shaded area in this graph represents the confidence interval around the regression line, which depicts the negative relationship between the R/S ratio and the 6-min walking distance. The wider interval, especially at higher R/S ratios, indicates greater variability in the data and less precision in predicting the walking distance as the R/S ratio increases. While the negative correlation is clear, the broader confidence interval suggests that the predictions become less certain at higher R/S ratios.

Additional parameters, such as QS duration in DI and aVL, showed non-significant correlations (Beta: −0.10, *p* = 0.681 for DI; Beta: −0.22, *p* = 0.330 for aVL), while the R/S ratio showed a significant negative relationship with walking distance (Beta: −16, *p* = 0.006), suggesting its potential relevance. An increase of this fraction by 1 was associated with a decrease in the walked distance of 16 m (Figure 10). Conversely, a composite parameter [(Sv1 + Rv6) − (Rv1 + Sv6)] did not demonstrate a significant correlation (Beta: −0.09, *p* = 0.449).

Overall, this detailed analysis provided valuable insights into the factors influencing physical performance as measured by the 6MWT. Statistically significant findings underscored the importance of specific clinical metrics, such as creatinine levels, NT-proBNP levels, diabetes, LVESV, systolic blood pressure, QRS duration, QRS area, and BIV pacing, in predicting exercise capacity. Further, the predictors that had an effect where the associated *p*-value was less than 0.05 were used for a univariate multiple linear regression (Table 2); then, using a backward selection algorithm, a model was constructed in which all predictors had statistically significant influences.

- Diabetes: The presence of diabetes is associated with a reduction of 44.65 m in walking distance, with a confidence interval (CI) ranging from −78.36 to −10.95 m (*p* = 0.010);
- QRS duration: Each 1 millisecond increase in QRS duration is associated with a decrease of 0.62 m in walking distance (CI: −0.81 to −0.44, *p* < 0.001);
- QRS area: Similarly, an increase of 1 square millimeter in the QRS area is associated with a 0.61 m reduction in walking distance (CI: −0.76 to −0.47, *p* < 0.001);
- R/S fraction: An increase in the R/S ratio is associated with a larger reduction in walking distance, with each unit increase resulting in a 20.29 m decrease (CI: −32.09 to −8.48, *p* = 0.001).

The random effects (σ^2^ = 473.02 and τ00 = 4758.94) suggest variability across individuals, and the intra-class correlation (ICC) of 0.91 indicates that a large proportion of the variance in walking distance can be attributed to differences between individuals. The model’s marginal R^2^ (0.336) indicates that the fixed effects (predictors) explain 33.6% of the variability in walking distance, while the conditional R^2^ (0.940) suggests that 94% of the variability is explained when both fixed and random effects are considered.

**Figure 11:**
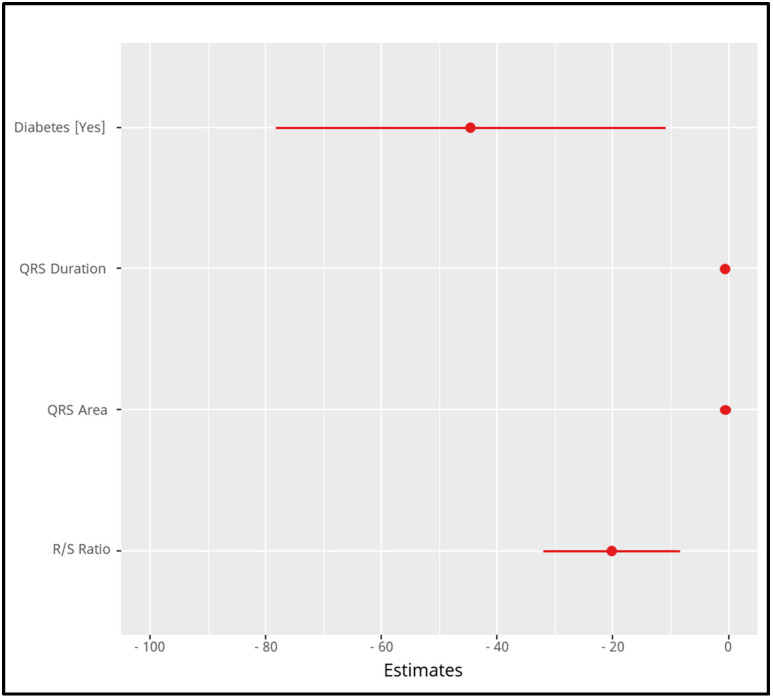
The plot presents the final model derived from a univariate multiple linear regression analysis. It shows the significant predictors of the six-minute walking distance, with estimates represented as red dots and the confidence intervals as horizontal lines. Having diabetes was associated with a negative effect on walking distance, with an estimate around −45, indicating that individuals with diabetes walked a shorter distance compared to those without diabetes. Each increase in QRS duration was associated with a reduction in walking distance, showing a strong negative correlation (steeper decline). Similar to QRS duration, a larger QRS area negatively affected the walking distance. The R/S ratio also showed a significant negative effect, with an increase in the R/S ratio leading to a substantial decrease in walking distance. All predictors shown in the model have statistically significant negative impacts on the walking distance, as their confidence intervals do not cross zero, reinforcing their influence on the reduction in the walking performance in the six-minute walk test.

Overall, the predictors that significantly influence walking distance are diabetes status, QRS duration, QRS area, and R/S ratio. These were included in a univariate multiple linear regression model (Figure 11), refined using a backward selection algorithm to ensure that all included predictors have statistically significant influences.

## 3. Discussion

Although multiple reference values for the six-minute walk distance (6MWD) in healthy adult populations have been proposed, their reliability remains uncertain due to significant methodological variations across studies. Several factors, including age, height, weight, sex, corridor length, cognitive impairment, and the need for continuous oxygen supplementation, have been shown to independently influence 6MWD outcomes in patients [13,14]. Consequently, these variables must be carefully considered when interpreting the results of the six-minute walk test (6MWT). Reported values for healthy subjects range between 400 and 700 m. Previous research has demonstrated a strong association between the six-minute walk distance (6MWD) and the functional status of patients with heart failure (HF), with the 6MWD also correlating with established metrics from cardiopulmonary exercise testing (CPET) [15]. Furthermore, the 6MWD offers an additional prognostic value beyond traditional measures. Research has shown a mild-to-moderate inverse correlation between functional status, as evaluated by the New York Heart Association (NYHA) classification, and 6MWD. A recent systematic review underscored this inverse relationship, reporting mean 6MWD values of approximately 400 m for NYHA class II, 320 m for NYHA class III, and 225 m for NYHA class IV patients with heart failure (HF). Notably, there was some overlap in 6MWD between NYHA class I and II patients, with both groups averaging around 400 m [15,16,17].

Patients with diabetes mellitus are at an increased risk of complications from arteriosclerotic heart disease and generally demonstrate lower levels of physical fitness compared to healthy adults. Additionally, studies have shown that individuals with diabetes mellitus exhibit a reduced cardiopulmonary capacity relative to non-diabetic subjects [18]. Research conducted by Kunitomi et al. on female patients with uncomplicated diabetes mellitus concluded that these individuals exhibited a smaller increase in heart rate and maximal oxygen uptake (VO_2_ max) during exercise compared to healthy subjects [19]. In our study, patients with diabetes mellitus walked an average of 33 m less than those without diabetes. A similar reduction in walking distance was observed for each single-unit increase in serum creatinine levels. Previous research has identified renal failure as a significant negative predictor of performance on the six-minute walk test (6MWT) and the perceived level of dyspnea. While 6MWT results are predictive of survival in dialysis patients, there are limited data on how modifiable factors related to dialysis therapy affect test outcomes. Comorbidities such as pulmonary disease, heart failure, peripheral arterial disease, or neurological conditions can also significantly reduce walking distance. However, the isolated impact of renal failure on 6MWT performance in dialysis patients with few or no comorbid conditions has yet to be thoroughly investigated [20].

Biventricular pacing enhances ventricular synchronization and improves cardiac efficiency, thereby reducing the frequency of heart failure exacerbations. This improved synchrony alleviates symptoms such as dyspnea and fatigue, enabling patients to engage in more physical activity [21]. Our study demonstrated that biventricular pacing increases walking distance by an average of 17 m for each percentage increase in pacing. To optimize the likelihood of favorable cardiac remodeling, the left ventricular electrode was positioned in the lateral, anterolateral, or posterolateral vein of the left ventricle, based on the widest Q-LV interval recorded for each patient. In ischemic patients, a cardiac MRI was employed to identify the most suitable vein for electrode placement, as this method has been shown to yield favorable outcomes [22].

QRS duration is a critical parameter for predicting the response to cardiac resynchronization therapy and has been incorporated into international guidelines to identify candidates who are most likely to benefit from this treatment [10]. Recent studies suggest that, compared to QRS duration, QRS area may be a superior predictor of outcomes following cardiac resynchronization therapy (CRT). According to the researchers, a larger QRS area has been shown to closely correlate with the delayed activation of the left ventricular (LV) lateral wall, independent of QRS morphology. There is a strong association between QRS area, clinical outcomes, and echocardiographic response. These findings indicate that QRS area may more accurately reflect the electrical substrate favorable for CRT and could serve as a criterion for identifying heart failure patients who are likely to benefit from this therapy [23].

Our analysis identified a correlation between QRS duration, QRS area, and walking distance. Patients with longer QRS durations walked 1.1 m less for each millisecond increase. Similarly, QRS area was negatively correlated with walking distance, with each 1 square millimeter increase in QRS area associated with a 0.94 m decrease in distance.

Sweeney et al. published a review proposing the activation pattern and R/S ratio as potential predictive parameters for outcomes following cardiac resynchronization therapy (CRT). A key aspect of their study was the morphological analysis of the QRS complex, dividing it into four possible waveform elements (R, S, Q, QS). Ventricular activation in each lead was characterized by nine possible patterns, referred to as QRS hieroglyphs: R, RS (R = S and both >1 mm or both <1 mm; equiphasic), Rs (R > S), rS (S > R), QS, qR (q < R), QR (Q = R > 1 mm or both <1 mm), Qr (Q > r), and QRS (all three waveforms present). The study concluded that these patterns were most pronounced in leads I, aVL, V1, and V2, which were designated as pivotal leads for predicting CRT outcomes [24]. Other researchers suggest that R/S variability in lead V1 may serve as a predictor of potentially incomplete biventricular pacing, which could negatively impact ventricular synchrony and exercise tolerance [25].

Our research revealed a negative association between the R/S ratio and the six-minute walking distance, with each 1-unit increase in this ratio corresponding to a reduction of 16 m in walking distance.

In a study by Guazzi et al., the authors suggest that there is a weak but significant correlation between left ventricular end-systolic volume (LVESV) and the results of the six-minute walk test [26].

Our study identified a negative correlation between LVESV and the distance traveled by the subjects. Specifically, each 1 mL increase in LVESV was associated with a median reduction of 0.30 m in the distance covered.

In our study, we included systolic blood pressure variation during the procedure to investigate its correlation with the outcome. This was based on the hypothesis that cardiac resynchronization therapy (CRT) would improve hemodynamic parameters, such as cardiac output, leading to an increase in systolic blood pressure. Previous research has explored this concept, with other authors establishing a 5 mmHg threshold for significant systolic blood pressure increase [27].

We adhered to this standard and found that an increase in monitored systolic blood pressure of more than 5 mmHg was positively correlated with walking distance, with a median increase of 38 m.

## 4. Study Limitations

Therapies like CRT, which involve cardiac remodeling, have an effect on a patient’s overall health, and multiple factors need to be taken into consideration. Regarding the observations and measurements needed to assess CRT therapy response, one should notice that optimization often occurs hours or days after implantation. The QRS duration may remain unchanged even six months post-treatment. These observations indicate time as a common denominator, and this is why timing should be considered when performing the 6 min walk test. The willingness of the subjects to perform the test and their ability to comprehend the instructions can also be a challenge. Some subjects may show a low motivation to participate, as heart failure is not only an organic affliction but also has an effect on their perception, mental health, and cognitive abilities.

About the number of subjects involved in this study, 69 patients is a relatively small number when speaking in absolute terms, but we consider that the relevance relies on the topics studied, the protocols used, and the quality of the observations that were made. None of our patients were included on any cardiac transplantation list. Although heart transplants are performed in our country, the number of transplant procedures is significantly lower than the demand.

## 5. Conclusions

To summarize, the 6 min walk test can provide a reliable view into a patient’s daily activity levels and short-term prognosis, particularly in those with heart failure and a reduced ejection fraction. It is inexpensive, easy to perform, and relatively less time-consuming compared to maximal exercise tests like cardiopulmonary exercise testing (CPET). Judging by our results and the correlations with well-established predictors, we noticed that the test can provide clinician insights into the prognostics of patients who have undergone cardiac resynchronization therapy. We think that future research should focus on a standardized methodology for this test and on providing more precise values to make more accurate predictions after cardiac resynchronization therapy.

## Figures and Tables

**Table 1:** The correlations of the main predictors with the 6MWD at 12 months. N is the number of measurements performed for the patients included in the study.

Characteristics	N	Beta (95% CI) ^1^	*p*-Value
Age	276	−0.98 (−2.7 to 0.77)	0.267
Gender			
Female	44	—	
Male	232	20 (−26 to 65)	0.391
Demographics			
Rural	112	—	
Urban	164	−23 (−57 to 10)	0.174
Hemoglobin	276	10 (−1.6 to 22)	0.091
Creatinine	276	−33 (−67 to −0.01)	0.05
NT-proBNP	276	−0.96 (−1.8 to −0.11)	0.028
Ischemia			
No	168	—	
Yes	108	−25 (−59 to 8.5)	0.141
Atrial fibrillation			
No	144	—	
Yes	132	−24 (−57 to 9.3)	0.156
Diabetes			
No	152	—	
Yes	124	−33 (−66 to −0.15)	0.049
LVESV	276	−0.30 (−0.54 to −0.06)	0.016
Sys blood pressure > 5 mmHg			
No	68	—	
Yes	208	38 (0.28 to 75)	0.05
QRS duration	276	−1.1 (−1.3 to −0.95)	<0.001
QRS area	276	−0.94 (−1.1 to −0.82)	<0.001
R amplitude in aVR	276	3.3 (−4.6 to 11)	0.405
R amplitude in right precordial leads	276	7.3 (−6.5 to 21)	0.294
BIV Pacing percentage	276	17 (0.33 to 34)	0.046
QS duration in DI	276	−0.10 (−0.60 to 0.39)	0.681
QS duration in aVL	276	−0.22 (−0.68 to 0.23)	0.330
R/S ratio	276	−16 (−27 to −4.6)	0.006
(Sv1 + Rv6) − (Rv1 + Sv6)	276	−0.09 (−0.33 to 0.15)	0.449

^1^ CI = confidence interval.

**Table 2:** The table presents the results of a six-minute walk test analysis, focusing on various predictors and their impact on walking distance. The intercept estimate is 474.88 m, indicating the baseline walking distance when no other factors are considered. Several predictors show a statistically significant effect on walking performance, as indicated by their *p*-values being less than 0.05.

	6 min Walk Test		
Predictors	Estimates	CI	*p*
(Intercept)	474.88	441.15–508.61	<0.001
Diabetes—yes	−44.65	−78.36–−10.95	0.010
QRS duration	−0.62	−0.81–−0.44	<0.001
QRS area	−0.61	−0.76–−0.47	<0.001
R/S fraction	−20.29	−32.09–−8.48	0.001
Random Effects			
σ^2^	473.02		
τ00 ID	4758.94		
ICC	0.91		
N ID	69		
Observations	276		
Marginal R^2^/conditional R^2^	0.336/0.940		

## Data Availability

Data are contained within the article.
